# Toxinotype V *Clostridium difficile* in Humans and Food Animals

**DOI:** 10.3201/eid1407.071641

**Published:** 2008-07

**Authors:** Michael A. Jhung, Angela D. Thompson, George E. Killgore, Walter E. Zukowski, Glenn Songer, Michael Warny, Stuart Johnson, Dale N. Gerding, L. Clifford McDonald, Brandi M. Limbago

**Affiliations:** * Centers for Disease Control and Prevention, Atlanta, Georgia, USA; † Hines Veterans Affairs Hospital, Chicago, Illinois, USA; ‡ University of Arizona, Tucson, Arizona, USA; § Acambis, Inc., Cambridge, Massachusetts, USA; ¶ Loyola University Chicago Stritch School of Medicine, Chicago

**Keywords:** Clostridium difficile, interspecies transmission, molecular epidemiology, research

## Abstract

*Clostridium difficile* is a recognized pathogen in neonatal pigs and may contribute to enteritis in calves. Toxinotype V strains have been rare causes of human *C. difficile*–associated disease (CDAD). We examined toxinotype V in human disease, the genetic relationship of animal and human toxinotype V strains, and in vitro toxin production of these strains. From 2001 through 2006, 8 (1.3%) of 620 patient isolates were identified as toxinotype V; before 2001, 7 (<0.02%) of ≈6,000 isolates were identified as toxinotype V. Six (46.2%) of 13 case-patients for whom information was available had community-associated CDAD. Molecular characterization showed a high degree of similarity between human and animal toxinotype V isolates; all contained a 39-bp *tcdC* deletion and most produced binary toxin. Further study is needed to understand the epidemiology of CDAD caused by toxinotype V *C. difficile*, including the potential of foodborne transmission to humans.

## Abstract 2

Such strains are uncommon causes of severe human disease but may be increasing in incidence.

Recent evidence suggests that the epidemiology of *Clostridium difficile*–associated disease (CDAD) is increasing in incidence and severity (*1*–*3*). These changes are due, at least in part, to the emergence of a more virulent *C. difficile* strain, designated NAP1 (based on its pulsed-field gel electrophoresis [PFGE] pattern), BI (by restriction endonuclease analysis [REA]), toxinotype III (by PCR characterization of the pathogenicity locus), and 027 (by PCR ribotyping) (*4*). However, the emergence of BI/NAP1/027 may not be solely responsible for changes in CDAD epidemiology, and the origin of this and other virulent strains is still largely unknown. *C. difficile* has also recently emerged as a pathogen or commensal in food animals such as neonatal pigs and beef and dairy calves (*5*–*7*); most of these animal isolates are toxigenic. Although several ribotypes have been identified in calves, the predominant ribotype in both calves and pigs is a toxinotype V strain (*8*,*9*). Moreover, recent reports suggest that *C. difficile* strains recognized as causes of human disease may contaminate retail meats (*10*).

To better understand whether food animals could be a source of infection for humans, we investigated recent and past human CDAD caused by toxinotype V *C. difficile* and compared isolates from these cases with those recovered from neonatal pigs and calves. We documented apparent changes in the frequency with which these toxinotype V strains cause human CDAD and compared the molecular characterization and toxin production of these strains with those of recent epidemic (i.e., BI/NAP1/027) and nonepidemic isolates.

## Materials and Methods

### Human Case Finding and Definitions

Case finding was performed by reviewing recent and past human isolates of interest from 2 sources. Cases were defined as patients with clinical isolates identified as toxinotype V by analysis of restriction fragment length polymorphisms (RFLPs) of toxin-encoding genes. First, we reviewed 620 *C. difficile* human isolates sent to the Centers for Disease Control and Prevention (CDC) from healthcare facilities and health departments in multiple states during hospital-associated outbreaks reported from 2001 through early 2007. Second, we reviewed a database of >6,000 isolates maintained by the Hines Veterans Affairs (VA) Hospital, representing CDAD reported from multiple healthcare facilities from 1984 up to January 1, 2001. All toxinotype V isolates were obtained from patients with a diagnosis of CDAD based on clinical history (e.g., diarrhea) and a positive clinical laboratory test for *C. difficile* toxin (e.g., cytotoxin assay or enzyme immunoassay).

Isolates identified from the Hines VA Hospital database were designated “past,” and isolates sent from hospitals to CDC from 2001 through early 2007 were designated “recent.” Additional clinical information was obtained for recent case-patients from a standard reporting form completed by the original submitting institutions. The proportions of past and recent isolates identified as toxinotype V were compared with the χ^2^ test results by using SPSS version 13.0 (SPSS, Inc., Chicago, IL, USA).

Case-patients were categorized with regard to likely place of acquisition according to recommendations developed by the *Clostridium difficile* Surveillance Working Group (*11*). Cases of CDAD were considered community-associated (CA-CDAD) if symptom onset (or positive *C. difficile* toxin test) occurred within <48 h after the patient was admitted to a healthcare facility, provided that it had been >12 weeks since the patient was last discharged from a healthcare facility. Cases of CDAD were considered healthcare facility–associated CDAD (HCFA-CDAD) if the patient had symptom onset >48 h after admission to a healthcare facility or was discharged from a healthcare facility within the previous 4 weeks. Cases were classified as indeterminate if symptom onset was within <48 h of a patient’s admission to a healthcare facility and 4–12 weeks since discharge from a previous admission. Case-patients were considered exposed to antimicrobial agents if the patient received a dose of any antimicrobial agent within the 30 days before symptom onset.

### Laboratory Methods

*C. difficile* isolates from humans and animals with clinical disease were obtained from diagnostic laboratories as previously described (*4*,*8*). Swine isolates were obtained from neonatal pigs with CDAD in North Carolina, Iowa, Texas, Utah, Ohio, and Arizona from 1999 to 2005. Bovine strains were isolated from January 1, 2003, through 2005, mainly from diarrheic Holstein calves 1 day to 6 weeks of age, originating in southern California, Arizona, New Mexico, Nevada, Texas, and Utah and maintained in pre-feedlot housing in calf ranches in Arizona. Domestic animal isolates were selected for study on the basis of host of origin, date of isolation, and geographic origin, all of which were independent. All food animal and human isolates were typed by PFGE with *Sma*I digestion as previously described (*12*,*13*) and analyzed with BioNumerics software version 4.01 (Applied Maths, Austin, TX, USA). Repeat PFGE analysis was performed with *Eag*I-digested DNA if human–food animal isolate pairs were indistinguishable when subjected to PFGE after digestion with *Sma*I or when *Sma*I digestion yielded too few bands for analysis. REA typing was performed, and patterns were compared as previously described (*12*). RFLP analysis of PCR fragments A3 and B1, from within *tcdA* and *tcdB*, respectively, was performed as previously described to determine toxinotypes (*14*).

PCR was used to detect *cdtB*, one of the genes encoding binary toxin. Deletions in *tcdC* were detected by PCR with primers tcdc1 and tcdc2, as described (*15*).

A subset of toxinotype V animal isolates (7 bovine, 7 porcine) and 7 recent human isolates were selected for toxin quantification. Production of toxins A and B was measured by ELISA as previously described (*1*). Toxin production was measured at 24 h, 48 h, and 72 h, and cell growth at 24 h and 48 h. Cell growth and in vitro toxin A and B production were compared between combined animal and human isolates of toxinotype V to human epidemic strain isolates (i.e., BI/NAP1/027; toxinotype III), and recent nonepidemic human strains (toxinotype 0) (*1*). Cell growth was compared by using the Student *t* test; the Mann-Whitney test was used to compare toxin production because toxin production values were not normally distributed.

Fourteen human (7 recent, 7 past) and 16 animal (8 bovine, 8 porcine) toxinotype V isolates were randomly selected for antimicrobial drug susceptibility testing. Susceptibility to clindamycin, levofloxacin, moxifloxacin, and gatifloxacin was determined by using E-test strips (AB Biodisk, Piscataway, NJ, USA) on Brucella agar plates with 5% sheep blood (Remel, Lenexa, KS, USA). Results were interpreted according to Clinical and Laboratory Standards Institute standard criteria (*16*). However, because no breakpoints have been established for levofloxacin and gatifloxacin, moxifloxacin breakpoints were used for interpretation of these results.

## Results

### Human Cases

Seven past cases of human infection with toxinotype V *C. difficile* were identified among the ≈6,000 human isolates in the Hines VA database; these cases occurred over 11 years before 2001. Eight additional recent cases were identified among the 620 human isolates sent to CDC from multiple states during 2001 through April 2006. The difference in proportions of past (<0.2%) and recent (1.3%) isolates that were toxinotype V was statistically significant (p<0.001). Three (38%) of 8 recent cases were CA-CDAD, 7 (88%) of such patients were exposed to antimicrobial agents, and 1 (13%) patient died from complications attributed to CDAD (Table 1). Four recent case-patients (50%) were male, and the median age was 71 years. Among patients for whom records were available, 3 (60%) of 5 past cases were judged to be CA-CDAD and 4 of 4 past cases were in persons exposed to antimicrobial agents.

**Table 1:** Clinical epidemiology of human toxinotype V *Clostridium difficile* cases, 1989–2006*

Origin	Location	Sex	Age, y	Antimicrobial agents†	Etiology	Diagnosis date	Disposition
1 R	Pennsylvania	F	75	Yes	HCFA	2001 May 2	Died
2 R	Illinois	M	54	Yes	CA	2003 Jul 24	Sent home
3 R	Iowa	F	71	Yes	HCFA	2004 Jul 29	Sent home
4 R	Texas	M	56	Yes	HCFA	2004 Nov 5	Sent home
5 R	Connecticut	F	85	Yes	HCFA	2004 Nov 21	Died
6 R	Georgia	M	72	Yes	CA	2005 Feb 8	Sent home
7 R	Connecticut	F	78	No	IND	2005 Jun 18	Sent home
8 R	Massachusetts	M	51	Yes	CA	2006 Jan 20	Died‡
1 P	Minnesota	M	NA	Yes	HCFA	1989 Apr 26	Unknown
2 P	Arizona	NA	NA	Unknown	Unknown	1991	Unknown
3 P	Illinois	M	71	Unknown	CA	1995 Mar 28	Unknown
4 P	Illinois	M	71	Yes	HCFA	1995 Apr 5	Unknown
5 P	Belgium	NA	NA	Unknown	Unknown	<1996	Unknown
6 P	Illinois	M	73	Yes	CA	1999 Sep 21	Sent home
7 P	Illinois	M	60	Yes	CA	1999 Nov 24	Sent home

*R, recent; P, past; HCFA, healthcare facility–associated; CA, community-associated; IND, indeterminate (patient with symptom onset <48 h of admission to healthcare facility and 4–12 wks since discharge from previous admission); NA, not available. †History of antimicrobial drug use within 30 d before *C. difficile*–associated disease (CDAD) diagnosis. ‡Death attributed to CDAD.

### Laboratory Results

**Figure 1:**
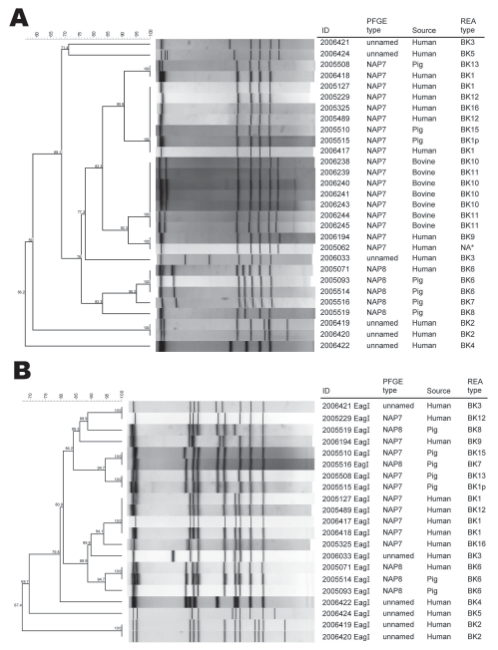
A). Dendrogram analysis of toxinotype V *Clostridium difficile* human and animal isolates using pulsed field gel electrophoresis (PFGE); *Sma*I restriction digest. Three animal-human isolate groups had indistinguishable PFGE patterns (2 NAP7 and 1 NAP8 group). Three of the NAP8 isolates (2005071, 2005093, 2005524) had identical REA types (BK6) as well. *PCR type unavailable. B) Dendrogram analysis using PFGE; *Eag*I restriction digest. One human–pig pair (2005071 and 2005514) had identical PFGE patterns by both *Eag*I and *Sma*I as well as identical REA patterns (BK6). Digestion of bovine isolates with *Eag*I yielded results that were not interpretable and were not included in this figure.

All 8 recent human isolates had a 39-bp deletion in *tcdC,* and 6 (75%) of 8 were binary toxin positive. All 7 past human isolates also had a 39-bp deletion in *tcdC,* and 7 (100%) of 7 were binary toxin positive. Thirty-three toxinotype V animal isolates were obtained; they displayed a variety of PFGE patterns (Figure 1). All, however, were binary toxin positive and had a 39-bp deletion in *tcdC*. Three animal–human isolate groups had indistinguishable PFGE patterns (100% similarity) when digestion was performed with the *Sma*I enzyme. The first group contained 1 human isolate (REA subtype BK1) that was indistinguishable by PFGE (NAP7) from 1 porcine isolate (REA subtype BK13). The second group consisted of 5 human and 2 porcine isolates, all of which were designated NAP7 by PFGE, although REA demonstrated 4 different subtypes. The third group contained 1 human isolate and 2 porcine isolates, which were indistinguishable by PFGE and REA (NAP8 and BK6). The 9 isolates in groups 1 and 2 were only 80% similar when digestion was performed with *Eag*I. However, the isolates in the third group were 95% similar, and 1 porcine isolate was indistinguishable (100% similarity) from 1 human isolate, even after digestion with *Eag*I.

**Figure 2:**
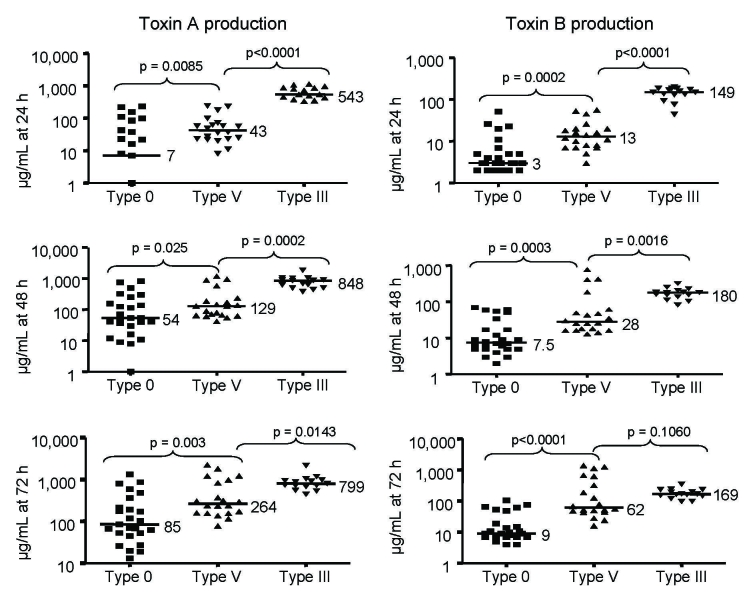
In vitro toxin production of toxinotype V *Clostridium difficile* isolates compared with epidemic toxinotype III and nonepidemic toxinotype 0 strains. Toxin A and Toxin B concentrations in micrograms per milliliter at 24, 48, and 72 h are shown for 25 toxinotype 0 isolates, 21 toxinotype V isolates (7 human; 14 animal), and 15 toxinotype III isolates. Horizontal lines indicate median values for each group and the p values are shown for comparison of the median toxin levels of toxinotype V isolates with toxinotype 0 and toxinotype III isolates.

Median toxin A and B production in the 21 toxinotype V isolates analyzed (7 bovine, 7 porcine, 7 of 8 recent human) was greater than that by nonepidemic toxinotype 0 isolates but less than that by epidemic toxinotype III isolates at all time points measured (Figure 2). The mean absorbance measurements at 600 nm, representing cell density, were measured at 24 h and 48 h and were not significantly different for toxinotype V isolates (1.56 and 1.06, respectively) than for toxinotype 0 isolates (1.77 and 1.39) or toxinotype III isolates (2.07 and 1.64).

Antimicrobial drug susceptibility testing was performed on 14 of 15 human and 16 of 33 animal toxinotype V isolates (Table 2). Resistance rates were similar overall in human and animal toxinotype V isolates except that more bovine isolates (88%) were susceptible to clindamycin than were porcine (0%, p<0.01) or human isolates (9%, p<0.01). All human and animal toxinotype V isolates (multiple strains by REA and PFGE) were susceptible to gatifloxacin and moxifloxacin, which differed markedly from human toxinotype III (BI/NAP1/027) and toxinotype 0 isolates (multiple strains by REA and PFGE).

**Table 2:** Antimicrobial drug susceptibility of toxinotype V *Clostridium difficile* isolates, 1989–2006*

Source	N	Clindamycin				Levofloxacin†				Moxifloxacin			Gatifloxacin†	
		S	I/R	Range		S	I/R	Range		S	Range		S	Range
Human	14	2 (14)	12 (86)	2–>256		6 (43)	8 (57)	2–>32		14 (100)	0.5–1		14 (100)	0.5–1
Recent	7	2 (29)	5 (71)	2–>256		0	7 (100)	>32		7 (100)	0.5		7 (100)	0.5–1
Past	7	0	7 (100)	4–>256		7 (100)	0	2–4		7 (100)	0.5–1		7 (100)	0.5–1
Porcine	8	0	8 (100)	4-–>256		2 (25)	6 (75)	2–>32		8 (100)	0.5–1		8 (100)	0.5–1
Bovine	8	7 (88)	1 (12)	1–4		8 (100)	0	2–4		8 (100)	0.5		8 (100)	0.5

*No. (%) *Clostidium difficile* isolates shown in each interpretive category. S, susceptible; I/R, intermediate or resistant. †Using moxifloxacin interpretive criteria.

## Discussion

In a review of recent and past isolates, we identified several human cases of CDAD caused by toxinotype V strains of *C. difficile*, which has been reported as a cause of epidemic disease in neonatal pigs and colonization in calves during the past decade (*9*,*17*,*18*). Moreover, different rates of occurrence in these temporally divergent populations suggest that toxinotype V may be an increasing cause of human CDAD, relative to other strains. The toxinotype V animal isolates included in our study have been previously identified as PCR ribotype 078, the most prevalent ribotype among calves and pigs, accounting for 94% (bovine) and 83% (swine) of isolates tested from multiple geographic regions (*8*). Food animal isolates we tested shared a high degree of similarity with human isolates, with 2 instances of animal–human isolate pairs appearing indistinguishable by REA or PFGE subtyping. In addition, all animal and human isolates displayed 39-bp deletions in *tcdC*, and most (45/47; 96%) were binary toxin positive.

Although *C. difficile* is recognized as a cause of disease in several animal species (*19*–*22*), little investigation has been conducted on the potential for interspecies transmission of *C. difficile* to humans. Previous studies have suggested the possibility of *C. difficile* transmission between humans and domestic pets (*23*,*24*), but no interspecies transmission has been documented, and few studies have examined the possible link between CDAD in food animals and humans. Identification of the same variant toxinotype strain as responsible for both human and animal disease in our study suggests at least 3 possible causes for human toxinotype V CDAD: 1) exposure of humans and animals to a common environmental source of *C. difficile*, 2) human disease caused by transmission by means of direct or indirect (e.g., through contaminated produce, water, or the environment) contact with infected live animals, and 3) human disease linked to consumption of products from food-producing animals. Both the genetic similarity of the human and animal isolates in our study and the apparent increasing importance of toxinotype V isolates in human CDAD after their emergence in animals may suggest foodborne or other forms of animal-to-human transmission.

In contrast to HCFA-CDAD, where patient-to-patient transmission of *C. difficile* is more likely, animal contact is a more plausible means of transmission for CA-CDAD. Our results suggest that toxinotype V *C. difficile* may be a relatively common cause of community-associated disease. Despite evidence that only 20% of all human CDAD cases are community-associated (*25*,*26*), 6 (46%) of 13 human toxinotype V cases in our study were identified as CA-CDAD. The high prevalence of CA-CDAD among toxinotype V cases we found is consistent with other studies that have identified variant toxinotypes more frequently in CA-CDAD than in HCFA-CDAD (*27*,*28*).

Toxinotype V strains may also be increasing as a cause of human CDAD since the emergence or recognition of epidemic toxinotype V disease in animals. In the past, reported frequencies of human strains with variant toxinotypes ranged from 6.4% to 13.4% of all *C. difficile* isolates collected (*29*–*33*), and toxinotype V strains contributed few cases to these frequency studies. However, a recent preliminary report from an Italian hospital indicated an upsurge in the proportion of binary toxin–positive *C. difficile* strains responsible for healthcare-associated disease in 2002 and 2003; most of these strains were toxinotype V (*34*). Toxinotype V appears to be an important cause of CDAD in food-producing pigs in parts of Europe, just as it is in North America (*35*).

The epidemiology of human CDAD has been affected by recent increases in the incidence and severity of disease. These changes have been largely attributed to the emergence of the BI/NAP1/027 *C. difficile* strain which, like the toxinotype V strains described here, is a toxin gene variant (i.e., toxinotype III) with an 18-bp deletion in *tcdC* (rather than the 39-bp deletion observed in toxinotype V strains) and with genes that encode binary toxin (*4*). In addition to this 18-bp deletion, and perhaps more importantly, BI/NAP1/027 has an upstream single nucleotide deletion at nucleotide position 117 (∆117), leading to a reading frameshift and early termination of protein translation (*27*).

Current literature suggests that this considerable truncation of TcdC may impair its negative regulatory function and contribute to the increased toxin production observed in BI/NAP1/027 strains (*27*,*36*). Molecular analysis of toxinotype V *C. difficile* has demonstrated a similarly truncated TcdC (61 aa compared with 65 aa in BI/NAP1/027 strains and 232 aa in wild-type TcdC) (*15*), which may imply hypervirulence for this strain as well. In contrast, isolates most commonly found in US hospitals before 2001 were toxinotype 0, had no polymorphisms in *tcdC*, and were binary toxin negative (*37*). Some of the increased virulence of BI/NAP1/027 may be due to its documented increased toxin A and B production in vitro (*1*). Although we did not find toxin production in toxinotype V isolates similar to BI/NAP1/027 levels, they did produce more toxin than nonepidemic toxinotype 0 isolates at all time points. Furthermore, the range of toxin A and B levels in our toxinotype V isolates was wide, and a minority produced toxin at similar or greater levels than BI/NAP1/027 strains.

This study is subject to the following limitations. First, the number of toxinotype V isolates examined was small and may not be wholly representative of this strain as it manifests in human or animal disease. Furthermore, recent isolates were collected from institutions that reported healthcare-associated outbreaks of CDAD, and clinical information describing patients from whom specimens were obtained may therefore overestimate disease severity. Moreover, since recent isolates were obtained from healthcare facilities that were experiencing CDAD outbreaks, they may represent a different population of patients than the past isolates, which were obtained from a variety of sources, some of which were ongoing clinical surveillance projects and some of which were outbreak investigations. The 2 source populations, however, can be considered reasonably similar in that both represent primarily hospitalized patients. If, however, the recent database contains a substantially greater proportion of outbreak-related isolates than the past collection, this would only strengthen the evidence for the recent emergence of toxinotype V CDAD. Since outbreaks of CDAD are largely associated with the epidemic, toxinotype III strain of *C. difficile* (*4*), relatively few toxinotype V isolates should be present in a recent database composed of outbreak-related isolates. The increased prevalence of toxinotype V in recent isolates compared with past ones may therefore represent an underestimate of the true prevalence of toxinotype V *C. difficile*.

Additionally, little is known about the types of *C. difficile* that cause disease in animals, which makes it impossible to determine whether the current toxinotype V strains are new or simply newly recognized. Finally, information about human cases is limited, particularly with respect to possible routes for community acquisition of disease; thus, evidence upon which to base conclusions regarding interspecies transmission is limited.

Although relatively common in animal CDAD, toxinotype V is currently an uncommon cause of human illness, which may occur more frequently among persons without traditional risk factors associated with CDAD, such as recent exposure to a healthcare setting. In vitro toxin production results from our limited sample suggest that toxinotype V strains have the potential to cause increased severity of human disease, although further studies are needed to corroborate this association. Although they share similar clinical features, evidence suggests that the predominant strains causing CDAD in humans and different animal species are distinct (*8*,*38*). Nonetheless, our finding of similarity between relatively widespread animal strains of *C. difficile* and strains responsible for occasional human disease raises the possibility of interspecies transmission. Further studies are needed to understand the etiology of CDAD caused by toxinotype V *C. difficile* and the mechanisms of transmission between animals and humans, including the role of the food supply.
